# Noncanonical outcomes of break-induced replication produce complex, extremely long-tract gene conversion events in yeast

**DOI:** 10.1093/g3journal/jkab245

**Published:** 2021-07-13

**Authors:** Joseph A Stewart, Michael B Hillegass, Joseph H Oberlitner, Ellen M Younkin, Beth F Wasserman, Anne M Casper

**Affiliations:** 1 Department of Environmental & Radiological Health Sciences, Colorado State University, Fort Collins, CO 80523, USA; 2 College of Dentistry, The Ohio State University, Columbus, OH 43210, USA; 3 Department of Biology, Interdisciplinary Graduate Program in Genetics, The University of Iowa, Iowa City, IA 52242, USA; 4 Department of Biology, Eastern Michigan University, Ypsilanti, MI 48197, USA

**Keywords:** loss of heterozygosity, LOH, gene conversion, LTGC, half crossover, gap repair, BIR, fragile site, homologous recombination, template switching, replication fork collapse, replication stress

## Abstract

Long-tract gene conversions (LTGC) can result from the repair of collapsed replication forks, and several mechanisms have been proposed to explain how the repair process produces this outcome. We studied LTGC events produced from repair collapsed forks at yeast fragile site FS2. Our analysis included chromosome sizing by contour-clamped homogeneous electric field electrophoresis, next-generation whole-genome sequencing, and Sanger sequencing across repair event junctions. We compared the sequence and structure of LTGC events in our cells to the expected qualities of LTGC events generated by proposed mechanisms. Our evidence indicates that some LTGC events arise from half-crossover during BIR, some LTGC events arise from gap repair, and some LTGC events can be explained by either gap repair or “late” template switch during BIR. Also based on our data, we propose that models of collapsed replication forks be revised to show not a one-end double-strand break (DSB), but rather a two-end DSB in which the ends are separated in time and subject to gap repair.

## Introduction

Replication fork collapse is a source of double-strand breaks (DSBs) in DNA. Regions that are difficult to replicate, such as fragile sites and repetitive sequences that form secondary structures, are particularly prone to DSB formation (Glover *et al.* 2017; Kim *et al.* 2017; Kononenko *et al.* 2018; Kaushal and Freudenreich 2019). Oncogene overexpression also increases replication fork stalling and collapse (Hills and Diffley 2014; Kotsantis *et al.* 2018; Tsao and Eckert 2018; Petropoulos *et al.* 2019), and the repair of DSBs at collapsed forks is hypothesized to contribute to genetic changes that drive the progression of cancer (Kotsantis *et al.* 2015; Tsao and Eckert 2018; Petropoulos *et al.* 2019).

During S phase and G2 of the cell cycle, homologous recombination is the favored DSB repair process because a sister chromatid or homologous chromosome can be used as a template for repair (Zierhut and Diffley 2008; Scully *et al.* 2019). If the sister chromatid is used as the template, the repair can be entirely conservative in restoring the broken DNA molecule. However, if the homologous chromosome is used as a template, the repair introduces alleles from the homolog onto the restored DNA molecule, resulting in loss of heterozygosity (LOH). This loss of alternate alleles is particularly important if a recessive mutant allele is uncovered within the region of LOH. The larger a region of LOH, the greater the potential to uncover a mutant allele that impairs cellular function, such as a mutant tumor suppressor.

Broadly, homologous recombination is divided into three sub-pathways: synthesis-dependent strand annealing, DSB repair, and break-induced replication (BIR; reviewed in Anand *et al.* 2013). The mechanistic detail of these pathways is well studied in both mammalian cells and yeast (Jasin and Rothstein, 2013; Wright *et al.* 2018). The canonical BIR pathway is engaged when the second end of the DSB is not available to participate in the repair process, such as in the case of a collapsed replication fork (Llorente *et al.* 2008; Kim *et al.* 2017; Kramara *et al.* 2018).

The canonical BIR process begins with 5′ end resection at the break, exposing a single-stranded 3′ end. Rad51p binds to the single-stranded region and facilitates a search for homology. The broken DNA invades a homologous template, forming a D-loop. This initiating invasion requires a significant region of homology (typically 200 bp or more) and very few mismatches are tolerated between the recombining strands (Lydeard *et al.* 2007; Anand *et al.* 2014). The invading 3′ end is extended by DNA synthesis through a noncanonical DNA replication process. The invading strand is prone to disengagement from the template (Smith *et al.* 2007). If the extended, invading 3′ end disengages from the D-loop during BIR, it can anneal to a different template. This process is termed “template switching,” and it is mechanistically different from the strand invasion that initiated BIR.

There are critical differences between template switch events and the initial invasion of a BIR. The initial strand invasion is much more strongly impeded by mismatches than template switching, and the initial invasion is much more dependent on Rad51p than template switching (Anand *et al.* 2014). During template switching, polymerase delta that was driving BIR is exchanged for the translesion polymerases pol zeta and Rev 1 (Sakofsky *et al.* 2015). Sakofsky *et al* (2015) also observed microhomologies and short inverted repeats at template switch junctions, similar to the copy number variation junctions reported in human DNA that are proposed to be Rad51-independent and reply on annealing to regions that are already single-stranded (Hastings *et al.* 2009).

Template switching in canonical BIR is restricted to within the first ∼15 kb of new DNA synthesis, after which the invading 3′ end is stabilized and the BIR replication fork becomes processive and can copy 100 kb or more of sequence (Smith *et al.* 2007; Mayle *et al.* 2015). The stabilized BIR fork is different from a typical replication fork because progression of replication occurs via migration of the D-loop with extrusion of nascent single-stranded DNA (ssDNA), and lagging strand synthesis is initiated sometime later on the extruded ssDNA (Saini *et al.* 2013; Sakofsky and Malkova 2017). The canonical BIR process ends after a telomere is copied.

When models of one-end DSBs (which are actually two-ended DSBs with homologous sequences present on only side) have been intentionally created, they are they are often repaired by canonical BIR that generates a large region of LOH extending from the site of invasion through the telomere (Anand *et al.* 2014; Sakofsky *et al.* 2014). The same outcome is reported from BIR repair of collapsed forks in cells under replication stress (Lemoine *et al.* 2005; Rosen *et al.* 2013; Zheng and Petes 2018). However, LOH extending through the telomere is not the only outcome reported from repair of collapsed forks; long-tract gene conversions (LTGC) have also been reported (Chumki *et al.* 2016; Hartlerode *et al.* 2016; Willis and Scully 2016). LTGCs are internal regions of LOH that are at least 15 kb long and can be 100 kb long or more (Yim *et al.* 2014; Chumki *et al.* 2016). LTGC events are more frequently observed in cells with defective BRCA1 or BRCA2, genes that are involved in hereditary breast and ovarian cancer (Chandramouly *et al.* 2011, 2013). Thus, LTGCs may be an important driver of tumor progression.

**Figure 1:**
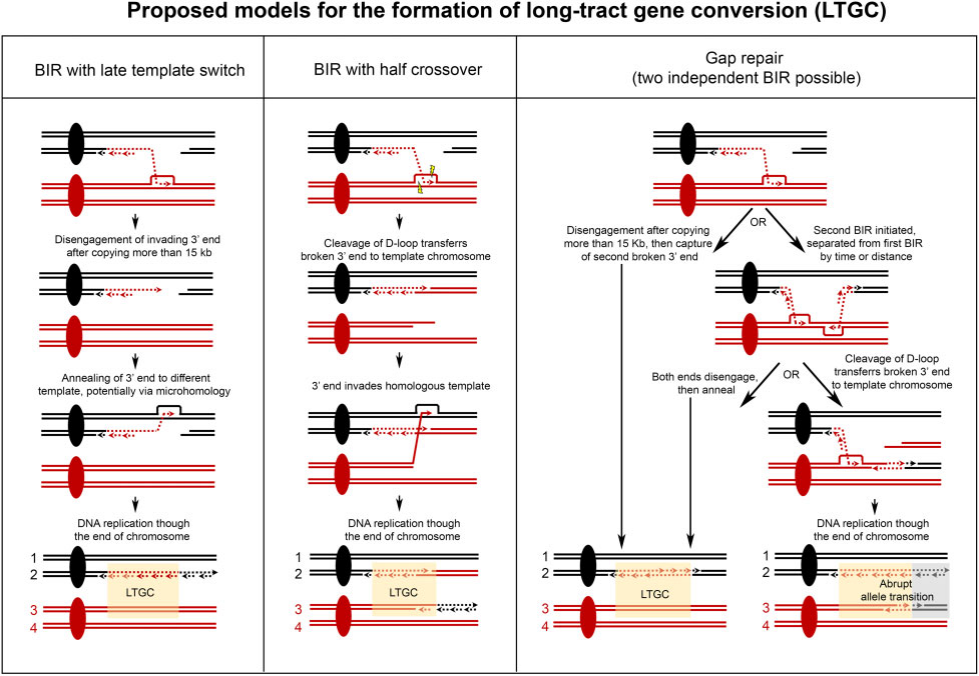
Proposed models for the formation of LTGC. A pair of homologous chromosomes is depicted, one gray and one red. Centromeres are indicated by filled-in circles. A double-strand break is shown on the gray homolog. In all of the proposed models, repair by homologous recombination is initiated when the broken gray chromosome and invades the homologous red chromosome, forming a D-loop. “BIR with late template switch”: The replication process is atypically interrupted, resulting in a noncanonical “late” disengagement of invading 3′ end after it has already been extended more than 15 kb. Interruption of BIR can result in annealing of the extended 3′ end to a region of microhomology, a process that is Rad51p-independent. If chromosomes 2 and 3 are segregated together during mitosis, then the cell with these chromosomes contains a LTGC. The SNPs flanking the gene conversion are in “coupling,” such that one chromosome III homolog has red alleles flanking both sides of the gene conversion region, and the other chromosome III homolog has gray alleles on both flanks. “BIR with half crossover”: Endonuclease cleavage of the D-loop transfers the break to the template chromosome. The broken template chromosome is repaired by a second BIR event. If chromosomes 2 and 3 are segregated together during mitosis, then the cell with these chromosomes contains a LTGC. The SNPs flanking the gene conversion are in “repulsion,” such that one chromosome III homolog has red alleles on the right side of conversion region and gray alleles to the left, and the other chromosome III homolog has the reverse. “Gap repair”: Either the second broken 3′ end is re-captured by the first end after it has extended more than 15 kb, or the second broken 3′ end initiates BIR independently of the first end. In the case of two independent BIR events, two possible resolutions are shown. Following the pathway on the left-hand side, both invading 3′ ends are extended, and then both disengage and anneal to each other. If chromosomes 2 and 3 are segregated together during mitosis, then the cell with these chromosomes contains a LTGC. The SNPs flanking the gene conversion are in “coupling,” similar to the outcome of late template switch. Following the pathway on the right-hand side, endonuclease cleavage of the centromere-distal D-loop transfers the break to the template chromosome. If chromosomes 2 and 3 are segregated together during mitosis, then the cell with these chromosomes contains an abrupt allele transition—that is, a region of homozygous red alleles followed immediately by a region of homozygous gray alleles.

It is unclear how LTGC events arise from repair of collapsed forks. Several models have been proposed for their formation as a noncanonical outcome of the BIR pathway (Figure 1; Voelkel-Meiman and Roeder 1990; Llorente *et al.* 2008; Lee *et al.* 2009; Smith *et al.* 2009; Chandramouly *et al.* 2013; Yin and Petes 2013; Vasan *et al.* 2014; Chumki *et al.* 2016; Willis and Scully 2016). LTGC might result from a noncanonical “late” disengagement of the 3′ end from the D-loop (*i.e.*, disengagement after more than ∼15 kb of synthesis), followed by template switch to the alternate homolog, a process that is likely to be microhomology-mediated. Or, LTGC might result from cleavage of the migrating D-loop, which produces a “half crossover” and transfers the initiating break to the chromosome being used as a template. Or, LTGC might result from gap repair in which the two ends of a DSB are separated in such a way that they initiate BIR independently of each other. We note that another potential outcome of the gap repair processes is a cell that after mitotic division has homologous chromosomes with an event that contains an abrupt transition between homozygous alleles (Figure 1).

Previously, we reported a surprising abundance of LTGC events from the repair of collapsed replication forks on yeast chromosome III at fragile site 2 (FS2) in a diploid model system (Casper *et al.* 2009; Rosen *et al.* 2013; Chumki *et al.* 2016). Here, we analyze in detail the molecular structure of these events and evaluate whether their characteristics are consistent with any of the proposed models of LTGC formation. Our analysis of LTGC events at FS2 entailed (1) evaluation of the sizes of the two chromosomes III in each diploid by contour-clamped homogeneous electric field (CHEF) gel electrophoresis and Southern blotting; (2) determination of the phasing of alleles on either side of each LTGC event by isolation of individual chromosome III homologs from a CHEF gel and testing of single-nucleotide polymorphisms (SNPs); (3) high-resolution definition of each LTGC event by Illumina next-generation whole-genome sequencing; and (4) detection of mutations at either end of each LTGC event by Sanger sequencing across junctions. We also used the next-generation sequencing data to characterize chromosomal changes genome wide in these strains.

Among 16 LTGC events, we report that 6 formed via half crossover during BIR, 3 formed via gap repair, and 7 could have formed by either gap repair or by “late” template switch during BIR. Some of the gap repair LTGC events we observed are highly complex and involve nonhomologous chromosomes. Altogether, we conclude that LTGC events result from a variety of noncanonical BIR outcomes.

## Materials and methods

### Experimental system to identify DNA repair events on yeast chromosome III

The LTGC repair events analyzed here were collected in our previous publication (Chumki *et al.* 2016). Our experimental system is a yeast diploid based on the design of Lee *et al.* (2009), which was created by mating together a YJM789-derived haploid (the “Y” haploid; Wei *et al.* 2007) with a haploid related to S288c (the “S” haploid). In our diploids, LOH near fragile site FS2 in a mitotic division at the time of plating results in a red/white sectored colony (Chumki *et al.* 2016).

**Figure 2:**
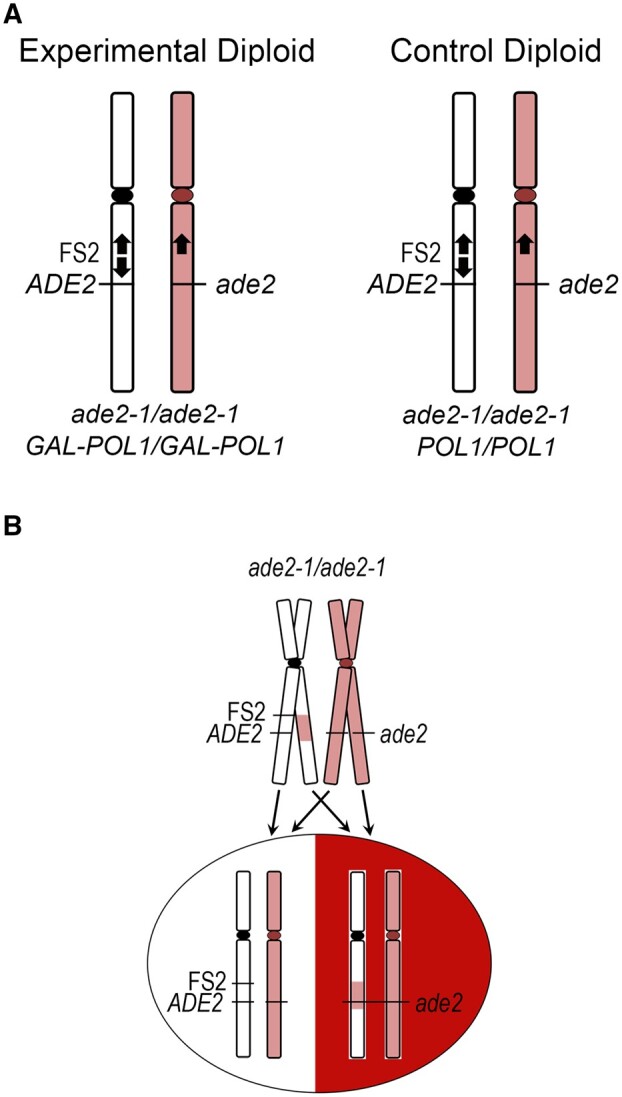
Yeast model system. The LTGC events analyzed here were collected from experimental diploid strains in our previous publication (Chumki *et al.* 2016). (A) The gray “S” homolog of chromosome III is related to S288c; the red “Y” homolog of chromosome III is related to YJM789. Ty1 elements are represented by black arrows. The experimental diploid is homozygous for *ade2-1* in its native genomic location. Fragile site FS2 is located on the “S” homolog of chromosome III. A full-length *ADE2* is inserted 150 bp centromere-distal to FS2, and a 5′ deletion allele of *ade2* inserted in the corresponding location on the opposite homolog. The experimental diploid is homozygous for the *GAL-POL1* construct that permits induction of replication stress by low levels of polymerase alpha. (B) Example of a LTGC resulting from repair of fragile site FS2 that causes LOH at the *ADE2/ade2* locus and produces a red/white sectored colony.

The experimental diploid cells in our model system from which LTGC events were collected are diagramed in Figure 2 (Chumki *et al.* 2016). These diploids are homozygous for the *ade2-1* allele in its native location on chromosome XV. This mutation is an ocher stop codon, and *ade2-1* yeast is red in color due to buildup of a red intermediate in the adenine biosynthetic pathway. The fragile site FS2 is hemizygous in our diploids. The S288C-related homolog of chromosome III carries the pair of Ty1 elements in inverted orientation that have been previously characterized as fragile site FS2 (Lemoine *et al.* 2005). The YJM789-related homolog of chromosome III has a single Ty1 element in this location, in Crick orientation. The experimental diploids have both copies of the *POL1* gene under control of the *GAL1/10* promoter (Chumki *et al.* 2016). The *POL1* gene encodes the catalytic subunit of yeast polymerase alpha primase. Cells grown in high-galactose medium (0.05%) have 300% of the normal amount of Pol1p, and those in low-galactose medium (0.005%) have 10% of normal Pol1p levels (Lemoine *et al.* 2005). The fragile site FS2 is unstable when cells are grown on the low-galactose medium, that is, when cells have low levels of polymerase alpha. Our diploid cells are also hemizygous for *ADE2* inserted 150 bp centromere-distal to fragile site FS2 on chromosome III from the “S” haploid; an *ade2* allele lacking its promoter and lacking the first 36 bases from the 5′ end is inserted at the corresponding location on chromosome III from the “Y” haploid.

In our previous publication (Chumki *et al.* 2016), we incubated cells in low-galactose medium to stimulate instability at FS2, then cells were plated for single colonies on medium with high galactose. After growth, gene conversion tracts crossing *ADE2* that form during a mitotic division at the time of planting result in a sectored colony (Figure 2). Loss of chromosome III, canonical BIR, or reciprocal crossover that cause LOH at *ADE2* in a mitotic division at plating also result in sectoring (Chumki *et al.* 2016). We fully describe in our previous publication how we used the zygosity of SNPs on chromosome III to classify each LOH event; in this publication we have further analyzed only the LTGC events collected previously.

### Growth media

All yeast strains were maintained at 30°C on standard rich media (Guthrie and Fink 1991). All media contained 3% raffinose instead of dextrose, because the raffinose carbon source does not affect the *GAL1/10* promoter. Galactose was added to the medium [no galactose, low galactose (0.005%), or high galactose (0.05%)] to control the *GAL1/10* promoter (Lemoine *et al.* 2005).

### CHEF gel electrophoresis

Genomic DNA from 1 × 10^8^ cells was harvested in agarose blocks to prevent shearing as described in Lobachev *et al.* (2002). Chromosomes were separated in a 1% gel in 0.5× Tris/Borate/EDTA (TBE) at 14°C using a Gene Navigator system (Pharmacia Biotech), or using a CHEF mapper system (BioRad). On the Gene Navigator, switch times to separate all yeast chromosomes were 6 V/cm, 50 s switch for 4.5 h, 90 s switch for 5.5 h, 105 s switch for 7.5 h, 124 s switch for 7.5 h, 170 s switch for 7.5 h. On the Gene Navigator, switch times optimized for separation of the “Y” and “S” homologs of chromosome III were 6 V/cm, 28 s switch for 6.5 h, 29.5 s switch for 6.5 h, 31 s switch for 6.5 h, 32.5 s switch for 6.5 h, 33.5 s switch for 6.5 h. On the CHEF Mapper, switch times starting at 47 s and extending to 2 min 49 s at 5 V/cm for 33 h were used to separate all yeast chromosomes. On the CHEF Mapper, settings for optimized separation of the “Y” and “S” homologs of chromosome III were: switch times starting at 26.5 s and extending to 58 s, switch time ramp a = −1.164, at 5 V/cm for 40 h. Gels were poststained with GelRed (Biotium). DNA in bands excised from CHEF gels was purified using the GeneJET gel extraction kit (Thermo Scientific).

### Southern blotting

DNA in CHEF gels was transferred to Hybond N+ membrane (GE Healthcare Life Sciences) by a neutral transfer according to standard protocol; then probed. Probes were made by PCR. Primers for the *CHA1* probe, a gene located on the left arm of chromosome III, were 5′-CTGGAAATATGAAATTGTCAGCGAC and 5′-TGAATGCCTTCAACCAAGTGGCTCCTTC. Primers for the *PAT1* probe, a gene located on the right arm of chromosome III, were 5′-AGGTGGTCAAGAACGAAACG and 5′-AGCCAATGGAATCTTTGTGG. Probes were biotin labeled using the North2South Biotin Random Prime Labeling Kit (Thermo Scientific). Probes were detected using the Chemiluminescent Nucleic Acid Detection Kit (Thermo Scientific). Images were captured with the ChemiDoc XRS+ imaging system (BioRad).

### SNP testing by PCR and restriction digest

The YJM789 and S288c strains differ in sequence by ∼0.5% (Wei *et al.* 2007), thus there are many SNPs throughout the genome of our experimental diploid. We characterize the zygosity of SNP by PCR to amplify polymorphic sites on chromosome III which change a restriction enzyme site. For example, a SNP on chromosome III at base 113,543 results in a *Mnl*I site on the YJM789-related chromosome but not on the S288c-related chromosome. This region was amplified by PCR, generating a 462 bp product. If the site is heterozygous in the cell being examined, digestion of the amplified product with *Mnl*I followed by gel electrophoresis reveals three band sizes: the uncut 462 bp product and the cut 335 and 127 bp products.

### Next-generation sequencing

We prepared DNA using the Nextera DNA Flex Library Prep Kit and Nextera DNA CD Indexes (Illumina). Next-generation sequencing and de-multiplexing were carried out by Novogene. We used CLC Genomics Workbench (QIAGEN Bioinformatics) to map reads to the *Saccharomyces* *cerevisiae* reference genome and detect SNP alleles. A table was exported from CLC that contained the location of each identified SNP in the genome, the percent of sequencing reads containing “Y” alleles at each SNP, and the number of sequencing reads crossing each SNP (*i.e.*, the depth of coverage). Microsoft Excel was used to plot the percent of reads that have “Y” alleles at each SNP, and to plot the number of sequencing reads crossing each SNP.

## Results

### Initial description of mitotic LTGC events at FS2

**Figure 3:**
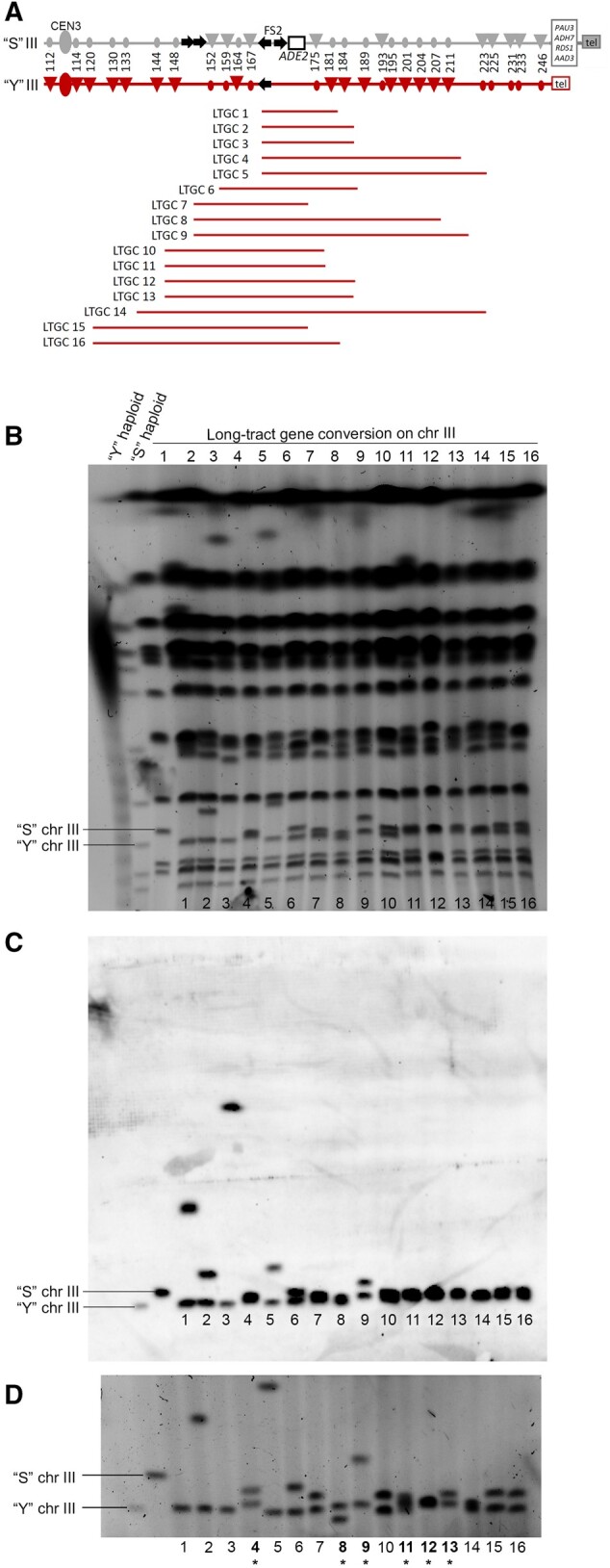
**Extent of LTGC events and sizing of chromosome III.** We evaluated 16 LTGC events on yeast chromosome III. (A) The gray “S” homolog of chromosome III is related to S288c; the red “Y” homolog of chromosome III is related to YJM789. Only the right arm of chromosome III is depicted. Ty1 elements are represented by black arrows, and centromeres are represented by ovals. The fragile site FS2 is only on the “S” homolog of chromosome III. SNP markers used to map repair events are shown by circles and triangles on the chromosome diagrams. Triangles indicate a restriction site exists, circles indicate lack of the site. Numbers are the approximate chromosome coordinate in kb. The LTGC are represented by horizontal lines. Line color indicates which homolog was copied during gene conversion. The initiation and termination of each gene conversion tract are depicted in the middle between the closest flanking SNPs but the actual location can be anywhere between the flanking SNPs. (B) DNA was extracted in agarose blocks and chromosomal DNA molecules were separated by clamped homogeneous gel electrophoresis as described in *Materials and Methods*. The initial sizes of the “S” and “Y” homologs of chromosome III are indicated. (C) The separated chromosome molecules were examined by Southern analysis using a probe to *CHA1*, which is located on the left arm of chromosome III. (D) CHEF gel with settings for optimized separation of the two homologs of chromosome III as described in *Materials and Methods*. The initial sizes of the “S” and “Y” homologs of chromosome III are indicated. The LTGC strains that contain a “Y” homolog that is, ∼6 kb larger than normal are indicated by a bold number with an asterisk below.

We previously reported that extremely LTGC events can occur as a result of repairing damage at fragile site FS2 in yeast diploid cells (Chumki *et al.* 2016). At the time, we characterized each mitotic repair event at FS2 using a set of 31 SNPs on yeast chromosome III (Chumki *et al.* 2016). Here, we further characterize in detail 16 strains that have LTGC resulting from repair at fragile site FS2. All of these mitotic LTGC events are longer than 15 kb, that is, they extend beyond the point at which the BIR replication fork becomes processive. The median length of LTGC in these 16 strains is 41.2 kb (range 15.4–86.5 kb; Figure 3A;Chumki *et al.* 2016). All of these LTGC events are heterozygous for SNPs tested outside the event and homozygous or hemizygous for SNPs tested within the gene conversion tract. Five of the events, LTGC 1 through LTGC 5, have one end of the gene conversion tract exactly at FS2. The other events, LTGC 6 through LTGC 16, likely had part of the 3′ ssDNA tail at the DSB degraded prior to strand invasion (Zierhut and Diffley 2008), because they have one end of the gene conversion tract centromere-proximal to FS2 and the other end of the tract is centromere-distal to FS2.

### Chromosome III sizes in cells with LTGC events across FS2

We determined whether the size of either homolog of chromosome III is altered in the diploid strains of our 16 LTGC events. We harvested genomic DNA from each strain in agarose blocks and separated chromosomes by CHEF electrophoresis (Figure 3B). We identified the two homologs of chromosome III by Southern blotting using a probe to *CHA1*, which is a gene located on the left arm of chromosome III (Figure 3, C and D).

The original sizes of the two homologs of chromosome III in our experimental diploid strain differ by ∼26 kb. The experimental diploid was initially created (Chumki *et al.* 2016) by mating together a YJM789-derived haploid (the “Y” haploid; Wei *et al.* 2007) with a haploid related to S288c (the “S” haploid). The original size of the “S” homolog of chromosome III is ∼346 kb, while the original “Y” homolog is ∼320 kb. The “Y” homolog of chromosome III is smaller because it contains fewer Ty elements on the right arm and because it lacks ∼9 kb at the end of the right arm; *PAU3* and all genes centromere-distal are located elsewhere in the YJM789-derived genome (Figure 3A; Supplementary Figure S1).

We cut out each chromosome III homolog from the CHEF gel and evaluated the SNP at base 113,543. This SNP is very close to the centromere on the left arm of chromosome III (the arm that does not contain the fragile site FS2). The allele present at this SNP was used to determine whether the cutout band was the “S” or the “Y” homolog of chromosome III. Based on this SNP analysis and on band sizing in the gel, we report that the size of the “Y” homolog of chromosome III is unchanged in ten of the 16 of the LTGC strains; the remaining six strains contain a “Y” homolog of chromosome III that is, ∼6 kb larger than the original size (Figure 3D and Table 1). None of the LTGC strains contain an “S” homolog of chromosome III that is the same size as the original. Instead, the “S” homolog of chromosome III is either larger (5 strains) or smaller (11 strains) than the original (Table 1).

**Table 1:** **In strains with LTGC on chromosome III, one or both chromosome homologs change size, and SNP phasing flanking the LTGC is variable**

Group placement	Strain name	Change in size of the “Y” homolog of chromosome III	Change in size of the “S” homolog of chromosome III	Phasing of heterozygous SNPs flanking the gene conversion
Coupling Group	LTGC 2	—	∼42 kb larger	Coupling
Coupling Group	LTGC 6	—	∼8 kb smaller	Coupling
Coupling Group	LTGC 7	—	∼15 kb smaller	Coupling
Coupling Group	LTGC 10	—	∼16 kb smaller	Coupling
Coupling Group	LTGC 14	—	∼20 kb smaller	Not determined*a*
Coupling Group	LTGC 15	—	∼13 kb smaller	Coupling
Coupling Group	LTGC 16	—	∼14 kb smaller	Coupling
Repulsion Group	LTGC 4	∼6 kb larger	∼9 kb smaller	Repulsion
Repulsion Group	LTGC 8	∼6 kb larger	∼36 kb smaller	Repulsion
Repulsion Group	LTGC 9	∼6 kb larger	∼12 kb larger	Repulsion
Repulsion Group	LTGC 11	∼6 kb larger	∼16 kb smaller	Not determined*a*
Repulsion Group	LTGC 12	∼6 kb larger	∼22 kb smaller	Not determined*a*
Repulsion Group	LTGC 13	∼6 kb larger	∼17 kb smaller	Repulsion
Disjoined Group	LTGC 1	—	∼204 kb larger	atypical*b*
Disjoined Group	LTGC 3	—	∼464 kb larger	atypical*b*
Disjoined Group	LTGC 5	—	∼66 kb larger	atypical*b*

a The two chromosome III homologs are too close in in size to be extracted individually from a CHEF gel for SNP phasing analysis.

b SNPs distal to the gene conversion tract from the “S” homolog of chromosome III homolog fail to be amplified by PCR.

### Phasing of alleles on either side of each LTGC event

Our previous method of analysis (Chumki *et al.* 2016) did not permit determination of the phasing of SNP alleles in the heterozygous regions flanking each gene conversion. Two different configurations are possible. The alleles may be in “coupling,” such that one chromosome III homolog has “Y” alleles flanking both sides of the gene conversion region, and the other chromosome III homolog has “S” alleles on both flanks. This is the expected configuration if the LTGC resulted from BIR with late template switch, or from gap repair (Figure 1). Alternatively, the SNPs flanking the gene conversion may be in “repulsion,” such that one chromosome III homolog has “Y” alleles on the right side of conversion region and “S” alleles to the left, and the other chromosome III homolog has the reverse. This is the expected configuration if the LTGC resulted from BIR with half crossover (Figure 1).

In 13 of our 16 LTGC strains, the two chromosome III homologs are different enough in size to be extracted individually from a CHEF gel for phasing analysis (Figure 3D). We used PCR and either restriction digest or Sanger sequencing to evaluate the SNPs on each individual homolog of chromosome III. For each homolog of chromosome III, we evaluated a SNP on the left arm near the centromere at base 113,543 (to confirm whether the particular band extracted is the “S” or “Y” homolog of chromosome III), and one or more SNPs around the gene conversion region (Supplementary Tables S1–S3).

Six strains, LTGC 2, 6, 7, 10, 15, and 16, have chromosome III SNPs in coupling flanking the gene conversion region (Table 1) and thus are consistent with formation of the LTGC either by BIR with late template switch, or by gap repair. Four strains, LTGC 4, 8, 9, and 13, have chromosome III SNPs in repulsion (Table 1) and thus are consistent with formation of the LTGC by BIR with half crossover. Three strains, LTGC 1, 3, and 5, were atypical, in that PCR fails to amplify SNPs distal to the gene conversion region on the “S” homolog of chromosome III (Table 1).

We note that all strains with SNPs in repulsion have a “Y” homolog of III is ∼6 kb larger than the original size (Figure 3D). The increase in size of the “Y” homolog is consistent with the mechanism of LTGC formation by BIR with half crossover, because the additional genes normally at the right telomere of the “S” homolog (*i.e.*, *PAU3* and all genes centromere-distal) would have been transferred by the half crossover onto the “Y” homolog. We did not specifically sequence the “Y” homolog, thus the increase in size could possibly be due to the inclusion of other DNA fragments.

### High-resolution definition of each LTGC event

**Figure 4.**
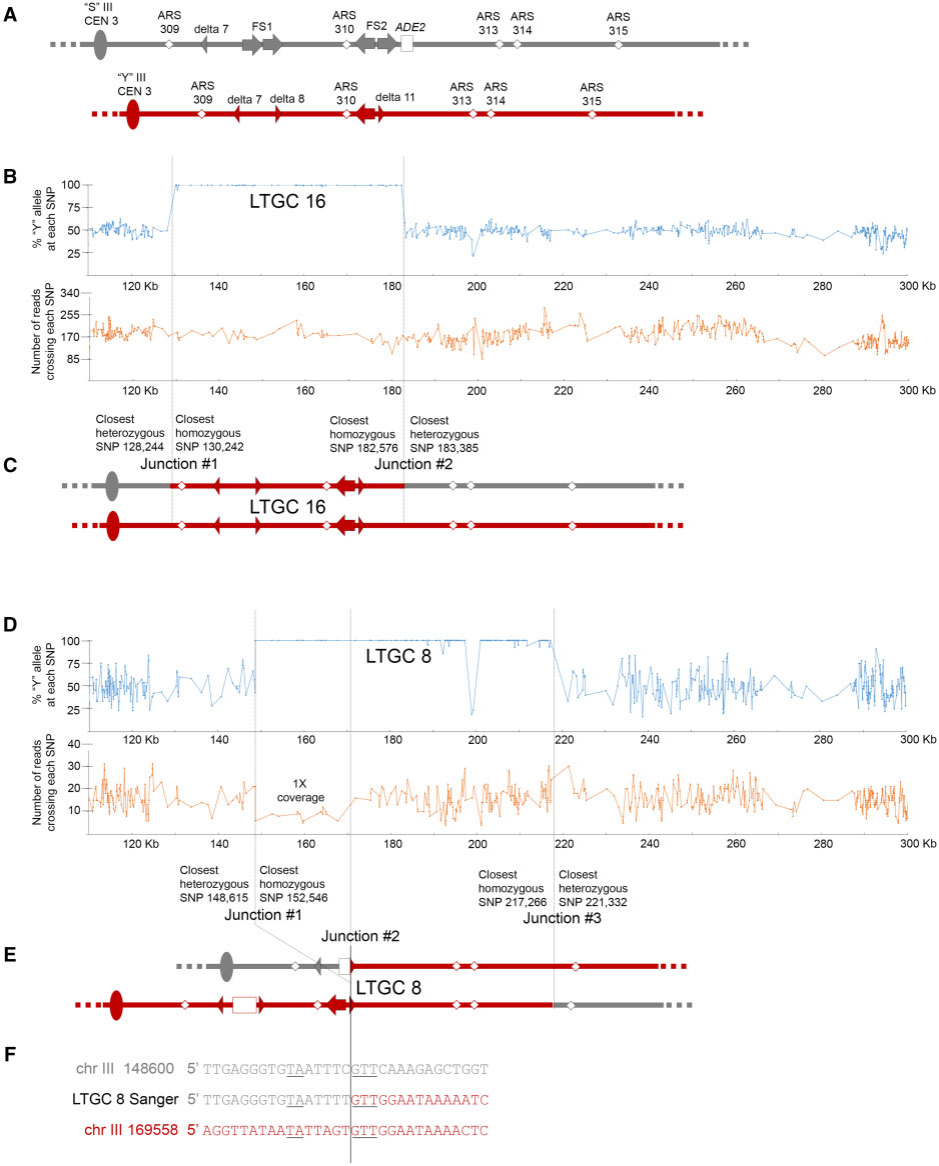
Analysis of next-generation sequencing data from two LTGC events. Blue graphs show the frequency of the “Y” allele at each SNP from next-generation sequecing. Orange graphs show sequencing coverage across each SNP. Dotted lines indicate junctions between SNPs that are heterozygous for both the “S” and “Y” alleles, and SNPs that are homozygous “Y” alleles. (A) Initial configuration of the two homologs of chromosome III in our experimental diploid. Only a portion of the right arm of chromosome III is depicted. The gray “S” homolog of chromosome III is related to S288c; the red “Y” homolog of chromosome III is related to YJM789. Origins are represented by white diamonds, delta elements are arrowheads, and Ty elements are arrows. (B) Whole-genome next-generation sequencing results from LTGC 16. Only data from the right arm of chromosome III is shown in the blue and orange graphs. The LTGC region appears as an expanse of SNPs in which 100% of the sequencing reads contain “Y” alleles. (C) Proposed configuration of the two homologs of chromosome III in LTGC 16, based on next-generation sequencing results and SNP phasing results. (D) Whole-genome next-generation sequencing results from LTGC 8. Only data from the right arm of chromosome III is shown in the blue and orange graphs. The LTGC region appears as an expanse of SNPs in which 100% of the sequencing reads contain “Y” alleles. (E) Proposed configuration of the two homologs of chromosome III in LTGC 8, based on next-generation sequencing results and SNP phasing results. (F) Sanger sequencing data of PCR product across deletion junction of LTGC 8. The original sequence of the “S” chromosome is shown in gray above, and the original sequence of the “Y” chromosome is shown in red below. Regions of microhomology are underlined.

The YJM789-related and S288c-related haploids that were mated together to form our experimental diploid differ in sequence by ∼0.5% (Wei *et al.* 2007). Thus, the 31 SNPs on chromosome III that we used previously to analyze LOH events near FS2 represent only a small fraction of the variants that exist (Chumki *et al.* 2016). Here, we used Illumina next-generation paired-end sequencing to analyze all SNPs throughout the entire genome of each of our 16 LTGC strains. We identified all informative SNPs through comparison of whole-genome sequencing of our two initial haploids. We then evaluated these SNPs in each LTGC strain. Our analysis of chromosome III from the next-generation sequencing of strains LTGC 8 and LTGC 16 is shown in Figure 4; all other LTGC strains are in Supplementary Figure S2.

At any SNP in the genome our experimental diploid cell, we expect that half of the sequencing reads will have the “S” allele and half of the sequencing reads will have the “Y” allele. However, when the broken “S” homolog of chromosome III uses the “Y” homolog as a template for repair, the DNA in the copied region is entirely “Y” allele SNPs. The edges of each gene conversion tract (which we call “junctions”) are between the last heterozygous SNP (*i.e.*, the last SNP with ∼50% “Y” alleles in the sequencing reads) and the first homozygous SNP (*i.e.*, the first SNP with 100% “Y” alleles in the sequencing reads). Our ability to pinpoint gene conversion tract junctions depends on the proximity of neighboring SNPs; typically, we can narrow each junction to a region < 4 kb in length. For example, our analysis of the junctions of the gene conversion tracts in strains LTGC 8 and LTGC 16 is shown in Figure 4.

In addition to mapping the edges of each LTGC tract with much higher resolution, we also assessed the depth of sequencing coverage to identify regions of deletion and duplication. In our experimental diploid cell, we expect the number of reads crossing each SNP located in unique sequence to be approximately equal throughout the genome. This average level of coverage we designate as “2× coverage.” For example, in LTGC 16 the number of sequencing reads crossing each SNP on right arm of chromosome III is steady (Figure 4B), and this depth of reads is consistent throughout the genome (data not shown), indicating 2× coverage. If one homolog of a chromosome is lost, or if part of one homolog is lost, then the number of sequencing reads crossing each SNP within the lost region drops by half (which we designate as “1× coverage”). For example, in LTGC 8 that there is a drop to 1X coverage on part of chromosome III (Figure 4D). If chromosome duplication has occurred, the number of sequencing reads within that region increases to 3× coverage.

### Groupings of LTGC events

**Figure 5:**
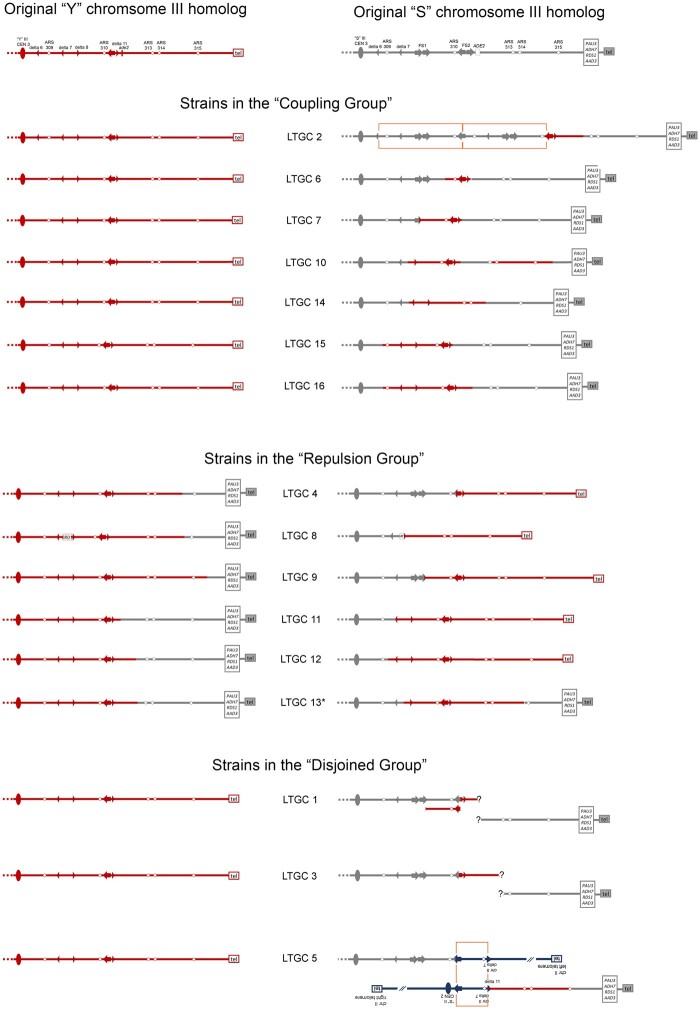
Strains with an LTGC event are divided into three groups. The configuration of the two original homologs of chromosome III in our experimental diploid is shown at the top. Only the right arm of chromosome III is depicted. The gray “S” homolog of chromosome III is related to S288c; the red “Y” homolog of chromosome III is related to YJM789. Each LTGC strain is shown with its homologs of chromosome III side-by-side, flanking the strain name.

Based the sizes of the two chromosome III homologs, the phasing of SNP alleles on each side of the gene conversion, and the next-generation sequencing results, we created three groupings of the LTGC strains (Table 2). A visualization of the features of strains in each grouping is shown in Figure 5, and Table 2 summarizes the possible explanations for each LTGC in the 16 strains analyzed.

**Table 2:** Summary of possible explanations for the LTGC on chromosome III in each strain analyzed

Group name	Strains included	Key shared characteristics	Possible mechanisms for LTGC formation
Coupling Group	LTGC 2 LTGC 6 LTGC 7 LTGC 10 LTGC 14 LTGC 15 LTGC 16	“Y” chromosome III is unchanged in size 2X sequencing coverage across chromosome III SNP alleles are in coupling on either side of the LTGC Size of the repaired “S” chromosome III observed on the CHEF gel is consistent with expectations based on the SNP mapping and sequencing coverage data	Either BIR with noncanonical late template switch, or gap repair
Repulsion group	LTGC 4 LTGC 8 LTGC 9 LTGC 11 LTGC 12 LTGC 13	“Y” chromosome III is ∼6 kb larger in size SNP alleles are in repulsion on either side of the LTGC (or phasing could not be determined)	BIR with half crossover
Disjoined group	LTGC 1 LTGC 3 LTGC 5	Repaired “S” chromosome III is larger than the original Sequencing coverage is consistently 2× across the entire chromosome III SNPs outside of the LTGC on chromosome III have ∼50% “S” and 50% “Y” alleles; Left and right arms of the “S” chromosome III are not together on the same chromosome	Gap repair with half crossover

The “Coupling Group” strains—LTGC 2, 6, 7, 10, 14, 15, and 16—share the following characteristics: (1) the “Y” homolog of chromosome III is unchanged in size; (2) there is 2× sequencing coverage across chromosome III; (3) on either side of the gene conversion tract, the SNP alleles are in coupling; (4) the size of the repaired “S” homolog of chromosome III observed on the CHEF gel is consistent with our expectation based on the SNP mapping and sequencing coverage data. There are some exceptions to these shared characteristics. Strain LTGC 10 has two LTGC regions on the right arm of chromosome III, but otherwise shares all the characteristics listed. Strain LTGC 2 has 3× sequencing coverage of a ∼40 kb region immediately centromere-proximal to the gene conversion (Supplementary Figure S2). Next-generation sequencing of the isolated “S” homolog of chromosome III confirms the presence of this amplification on that homolog (data not shown). Otherwise, LTGC 2 shares the characteristics of this group. Strain LTGC 14 has 1× sequencing coverage of a ∼20 kb region within the gene conversion region on chromosome III (Supplementary Figure S2). The size of the repaired “S” chromosome III on Southern blot is consistent with deletion of this 1× coverage region (Figure 3C). The two chromosome III homologs are approximately the same size in LTGC 14, thus we are unable to separate individual homologs for further analysis, but the other characteristics of this strain are consistent with the characteristics of this group.

Overall, the “Coupling Group” strains are consistent with LTGC formation by either BIR with noncanonical late template switch or gap repair (Figure 1). If a LTGC was formed by late template switch, sequence alteration could be present at the switch junction. This is because template switching is tolerant of mismatches between the broken 3′ end and the homologous template (Anand *et al.* 2014) and may be Rad51p-independent (Ira and Haber 2002). Also, in *pif1* mutant yeast cells where BIR is always interrupted, more than half of template switch junctions contain sequence alterations indicative of microhomology-mediated recombination (Sakofsky *et al.* 2015). In mammalian cells, complex breakpoints suggestive of microhomology-mediated recombination are frequently observed at sites of Noncanonical BIR termination (Hartlerode *et al.* 2016). We designed PCR primers to amplify LTGC event junctions that were not at a Ty element (Supplementary Table S2). The location of each junction is indicated in Figure 4 and Supplementary Figure S2. PCR was done on chromosome III homologs isolated individually from CHEF gels, and the products were analyzed by Sanger sequencing. We compared the junction sequences to the original sequence of the “S” and “Y” homologs of chromosome III. None of the “Coupling Group” strains contain junction sequence that differs from the original sequence. Although we did not find any sequence alterations at the junctions, this does not rule out late template switch as a possible mechanism. Thus for the “Coupling Group” strains, either gap repair or late template switch could be mechanism for formation of LTGC.

The “Repulsion Group” strains—LTGC 4, 8, 9, 11, 12, and 13—share the following characteristics: (1) they all contain a “Y” homolog of chromosome III is ∼6 kb larger in size, and (2) on either side of the gene conversion tract, the SNP alleles are in repulsion (or phasing could not be determined; Table 1 and Supplementary Figure S2). The increase in size of the “Y” homolog and the SNPs in repulsion are consistent with LTGC formation by BIR with half crossover. We designed PCR primers for all “Repulsion Group” LTGC junctions that were not at a Ty element (Supplementary Table S2), and amplified each junction from chromosome III homologs isolated individually from CHEF gels. Sanger sequencing and analysis of the PCR products demonstrated that none of these junctions, save one, differ from the original sequence.

The sole LTGC junction that differs from the original sequence among the “Repulsion Group” strains is within LTGC 8. In this strain, a portion within the LTGC region on chromosome III is 1× coverage in the whole-genome sequencing data (Figure 4D), and the reduction in size of the repaired “S” chromosome III on Southern blot is consistent with deletion of this 1× coverage region (Figure 3C). Next-generation sequencing of the isolated “S” homolog of chromosome III in LTGC 8 confirms that there is little or no sequencing coverage in the deleted region (Supplementary Figure S3). Sanger sequencing of the PCR product across the deletion junction reveals there are three bases of microhomology in the deletion junction, as well as a nearby two-base island of microhomology (Figure 4F). This data suggests that in LTGC 8, BIR was initiated, followed shortly by a microhomology-mediated template switch, and later, a half crossover generated the gene conversion.

The “Disjoined Group” strains—LTGC 1, 3, 5—have a repaired “S” homolog of chromosome III that is larger than the original (Figure 3C). However, these strains have sequencing coverage that is consistently 2× across the entire chromosome III, and SNPs outside of the LTGC on chromosome III have ∼50% “S” and 50% “Y” alleles (Supplementary Figure S2).

Initially, we had used a probe to the *CHA1* gene on the left arm of chromosome III for Southern blot to identify chromosome III (Figure 3C). We carried out an additional Southern blot, this time using a probe to the *PAT1* gene on the right arm of chromosome III. In the three “Disjoined Group” strains, the *PAT1* probe hybridizes to different bands than the *CHA1* probe (Table 3 and Supplementary Figure S4); in all other LTGC strains the *PAT1* and *CHA1* Southern results are identical. Thus, in LTGC 1, 3, and 5, the left and right arms of the “S” homolog of chromosome III are not together on the same chromosome. Yet, whole-genome sequencing indicates both the Y homolog and the S homolog of chromosome III are completely present within the genome of these three strains.

**Table 3:** Probes to the left and right arms of chromosome III detect different band sizes on Southern blot

Strain name	*CHA1* probe (left arm of chromosome III)	*PAT1* probe (right arm of chromosome III)
LTGC 1	320 and 550 kb	320 and 930 kb
LTGC 3	320 and 810 kb	320 and 1,300 kb
LTGC 5	320 and 412 kb	320 and 780 kb

Because the left and right arms of the “S” chromosome III are not together in LTGC 1, 3, and 5 (Figure 5), we propose that the two sides of the break independently initiated BIR events—one invading the “Y” chromosome III homolog, and the other invading a nonhomologous chromosome. In order for the broken right arm of the chromosome III to be retained in the cell, the BIR event that is initiated by this arm is likely to be resolved through a half crossover such that it becomes attached to a chromosome containing a centromere. Thus, a mechanism involving both gap repair and half crossover can explain the “Disjoined Group” strains LTGC 1, 3, and 5. Further detailed analysis of strain LTGC 5 is consistent with this proposed mechanism (Supplementary Figure S5).

### Unselected events genome-wide in strains with a LTGC on chromosome III

In our experimental system, we lower the level of polymerase alpha in our yeast cells to cause replication stress, which causes breaks at fragile site FS2 on yeast chromosome III (Lemoine *et al.* 2005; Chumki *et al.* 2016). However, this stress is likely to also cause breaks at other replication forks throughout the genome. As expected, our next-generation whole-genome sequencing data reveals other genomic changes in addition to the LTGC on chromosome III in these strains. Among our 16 strains, we observed 8 copy number variants, and 4 short-tract gene conversions on other chromosomes (Table 4). There are also 15 events that are either canonical BIR or reciprocal crossover; these cannot be distinguished from one another because we did not carry out whole-genome sequencing on both sides each sectored colony, and because they could have occurred in a cell division prior to the division that resulted in red/white sectoring on the screening plate.

**Table 4:** Unselected events in strains with a LTGC on chromosome III are detected by whole-genome next-generation sequencing

Strain name	BIR or reciprocal crossover	Copy number variant (CNV)	Gene conversion
LTGC 1	Chr V right arm starting at ∼355 K*a*	–	Chr X ∼101-107 K
LTGC 2	Chr XII right arm starting at ∼382 K*a*	—	—
LTGC 3	Chr VII left arm stating at ∼269 K	—	—
	Chr XII right arm starting at ∼450 K		
LTGC 4	–	Chr II amplification of ∼221-224 K	—
		Chr XV amplification of ∼849-851 K	
LTGC 5	Chr XIV left arm starting at ∼263 K	Chr II amplification of ∼197-224 K*b*	Chr IV ∼1333-1339 K
LTGC 6*c*	—	—	—
LTGC 7	—	—	—
LTGC 8	Chr XII right arm starting at ∼599 K	—	—
	Chr XV right arm starting at ∼710 K*a*		
LTGC 9	—	—	—
LTGC 10	Chr IV right arm starting at ∼910 K	Chr XV amplification of ∼849-851 K	Chr IV ∼378-413 K
	Chr XIII left arm starting at ∼165 K*a*		
	Chr XVI left arm starting at ∼416 K		
LTGC 11	Chr VIII right arm starting at ∼387 K*a*	Chr IV deletion of ∼1156-1164 K	—
		Chr XII deletion of ∼599-651 K	
LTGC 12	Chr XII right arm starting at ∼450 K	—	—
	Chr XVI right arm starting at ∼929 K		
LTGC 13	—	—	Chr XV ∼628-634 K
LTGC 14	Chr XIII right arm starting at ∼281 K	Chr XV amplification of ∼849-851 K	—
LTGC 15	—	Chr IV deletion of ∼1201-1212 K	—
LTGC 16	Chr XIV left arm starting at ∼345 K	—	—

a This event contains abrupt transition between homozygous alleles, as in the right-hand pathway of gap repair in Figure 1.

b This CNV is associated with the LTGC on chromosome III (see Supplementary Figure S5).

c This strain had low sequencing coverage and thus unselected events may have been missed in our analysis.

We highlight that 2 of the 15 unselected BIR or reciprocal crossover events begin in the rDNA array on yeast chromosome XII (∼450 K). We previously reported that the rDNA array is particularly unstable in cells under replication stress from low polymerase alpha (Casper *et al.* 2008). We also note that three of the eight copy number variation events are duplication of the region between *RDL1* and *RDL2* on chromosome XV (∼849–851 K); these genes share significant homology. All of the other unselected events that we observed are unique to their strain.

### Homologous chromosomes that have an abrupt transition between homozygous alleles

**Figure 6:**
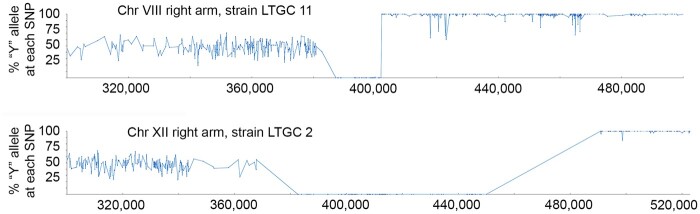
Examples of events with abrupt allele transition. Blue graphs show the frequency of the “Y” allele at each SNP from whole-genome next-generation sequencing. In each graph, the event at first shows all of the sequencing reads having the “S” allele. Later in the event, there is an abrupt allele transition is such that all the sequencing reads have the “Y” allele. The amount of sequencing coverage across the chromosomal regions shown remains constant (not shown). In strain LTGC 11 on right arm of chromosome VIII, the transition from “S” to “Y” is at *EGT2*. In strain LTGC 2 on right arm of chromosome XII, the transition from “S” to “Y” is within the rDNA array.

As noted in the proposed models for the formation of LTGC, a possible outcome of the gap repair mechanism is a cell that after mitotic division contains a pair of homologous chromosomes with an event that contains an abrupt transition between homozygous alleles (Figure 1). Among the 15 unselected BIR or reciprocal crossover events, 5 (33%) have an abrupt transition between homozygous alleles (Table 4); 2 examples are shown in Figure 6. As expected, outside of the event, half of the sequencing reads have “Y” SNP alleles and half have “S” alleles, and at the start of the event, all sequencing reads have the same allele (either all “Y” or all “S”). However, at a point within the event there is an abrupt transition, such that all the sequencing reads have the opposite allele. Despite this switch, the amount of sequencing coverage remains constant.

## Discussion

Here, we report the detailed characteristics of 16 extremely LTGC events in the yeast *S.* *cerevisiae*. The events we examined were previously collected through a screen for LOH resulting from repair of replication-stress induced lesions at fragile site FS2 on yeast chromosome III (Chumki *et al.* 2016). We observed evidence specifically supporting LTGC formation via half crossover during BIR for six events, and evidence specifically supporting LTGC formation via gap repair for three events. The remaining seven events have characteristics consistent with either LTGC formation by gap repair or from “late” template switch during BIR. Overall, multiple types of noncanonical BIR outcomes are responsible for LTGC events.

We observed that 6 of our 16 LTGC events at fragile site FS2 have characteristics that are consistent with half crossover resolution of a BIR event (Figures 1 and 5 andTable 2). Although half-crossovers have been previously reported to resolve BIR events, these data come from cells that lack Pol32p, an essential protein for BIR, of from *pif1* mutant cells, or from cells exposed to the DNA damaging agents MMS or 4-NQO (Llorente *et al.* 2008; Sakofsky *et al.* 2015; Kramara *et al.* 2018). In these previous reports of half crossover BIR resolution, the tract of DNA synthesis is very short, indicating that the D-loop is cleaved soon after strand invasion by the 3′ end. In contrast, our data indicate that BIR events can be resolved by half-crossover resolution can have extensive tracts of recombination-initiated DNA synthesis prior to the half crossover.

Seven of the LTGC events that we studied in detail have characteristics that are consistent with either noncanonical “late” template switch during BIR, or with gap repair. We attempted to determine whether late template switch was a more favorable explanation by looking for sequence alterations across gene conversion junctions in these strains. Sequence alterations are possible at template switch events, because even in yeast strains with wild type *RAD51*, Anand *et al.* (2014) detected patches of mismatches that went uncorrected during template switch, and Sakofsky *et al.* (2015) observed microhomologies and short inverted repeats at template switch junctions. We did not observe any sequence alterations at the junctions among these seven strains. In the absence of evidence to support a particular mechanism, both gap repair and late template switch are possible for LTGC formation in these strains.

Three of our LTGC events contain a disjoined chromosome III—the left and right arms of the chromosome, while both present in the cell, are not together. This finding indicates that the LTGC resulted from two independent BIR events, that is, gap repair. Fragile site FS2 consists of an inverted pair of retrotransposons (Lemoine *et al.* 2005), thus while one broken end initiated BIR using the homologous chromosome III as a template, the other broken end initiated BIR using a repetitive element in a nonhomologous chromosome. Normally, BIR is restrained at a two-ended DSB by coordinating the simultaneous engagement of both ends of the break, through Rad52p or Rad59 annealing of the second broken end, and through synchronous resection of both broken ends and possibly tethering by the Mre11-Rad50-Xrs2 (MRX) complex (Pham *et al.* 2021). However, the simultaneous engagement of broken ends that limits BIR would be precluded by significant temporal separation in when the two ends are available for repair. Thus, a time lag between the two broken ends at FS2 would permit thus the two sides of the break independently initiate BIR (gap repair; Figure 1). We suggest that such a time lag is possible through replication dynamics on yeast chromosome III. Fragile site FS2 is located ∼1kb from replication origin ARS 310, and this origin fires relatively early in 90% of cell cycles (Poloumienko *et al.* 2001). On the other side of FS2, the nearest highly efficient replication origin is ARS 315, located ∼55 kb centromere-distal of the fragile site. Thus, if a replication fork originating from ARS 310 collapses to a one-end DSB at FS2, there is likely to be a substantial time lag before a converging fork from ARS 315 generates the second end of the DSB.

Finally, we highlight the finding that among unselected events genome-wide in cells under replication stress, five have an abrupt transition between homozygous alleles within the event. This outcome may be a signature of a mechanism involving both gap repair and half-crossover (Figure 1). Further studies are needed to evaluate the frequency of these abrupt allele transitions and further understand them.

We propose that the traditional model of a collapsed replication fork (reviewed in Llorente *et al.* 2008; Kramara *et al.* 2018) be expanded; instead of generating a one-end DSB, a collapsed fork can generate a two-end DSB in which the ends are separated in time. Homologous recombination at collapsed forks and BIR in particular are highly relevant to the genetic changes involved in tumor initiation and progression. This expanded model will prompt clearer data evaluation in light of likely gap repair at collapsed replication forks.

## Data availability

Strains and plasmids are available upon request. Supplementary files available at the GSA Figshare portal: https://doi.org/10.25387/g3.14689173. Supplementary Figure S1 is *PAU3* and other genes missing from the “Y” homolog of chromosome III. Supplementary Figure S2 is all LTGC strains not in the main manuscript. Supplementary Figure S3 is sequencing of the isolated “S” homolog of chromosome III in strain LTGC8. Supplementary Figure S4 is a Southern Blot probed with *PAT1*. Supplementary Fig S5 is additional details from strain LTGC 5. Supplementary Table S1 is primers used to test SNPs. Supplementary Table S2 is primers used to amplify junctions. Supplementary Table S3 is the SNP analysis data used for phasing of alleles around each LTGC. Sequencing reads have been deposited and are publicly available at https://www.ncbi.nlm.nih.gov/sra/PRJNA744506.

## Funding

J. Stewart and M. Hillegass were supported by an Honors Program Research Fellowship at Eastern Michigan University (EMU). J. Stewart was also supported by the EMU Undergraduate Research Stimulus Program. This research was supported by National Institutes of Health grant 1R15GM124651-01 to A.M.C.

## Conflicts of interest

None declared.
